# Attitudes and Determinants Affecting Telehealth and Telemedicine Use in the MENA Region: A Narrative Review

**DOI:** 10.3390/healthcare14162442

**Published:** 2026-08-07

**Authors:** Sneha Reji, Mayssah El Nayal, Jayadevan Sreedharan

**Affiliations:** 1 Department of Family & Community Medicine and Behavioral Sciences, College of Medicine, University of Sharjah, Sharjah P.O. Box 27272, United Arab Emirates; snehareji2021@gmail.com; 2 College of Medicine, Gulf Medical University, Ajman P.O. Box 4184, United Arab Emirates; 3 Student Wellbeing Center, Gulf Medical University, Ajman P.O. Box 4184, United Arab Emirates; 4 Thumbay Institute of Population Health, Gulf Medical University, Ajman P.O. Box 4184, United Arab Emirates

**Keywords:** telehealth, telemedicine, digital health

## Abstract

**Background/Objectives**: Telehealth (TH) and Telemedicine (TM) can significantly benefit individuals who live far from healthcare institutions and those with limited financial resources by improving access to essential health services. This review explores the attitudes and determinants affecting the use of TH and TM, focusing on the MENA region with insights from the global context. **Methods**: A narrative review was created using a compilation of scientific articles published between 2020 and 2025 from PubMed and Scopus. Studies were screened to be relevant with the determinants, attitudes or use of TH and TM. Forty one sources met the eligibility criteria and covered evidence from the MENA region with supporting background from global developments. **Results**: In the MENA region, although high perceived effectiveness is recorded, the use of TH and TM remains below optimal levels. The majority of the studies reviewed suggested that participants have positive attitudes towards TH and TM. Nevertheless, some studies suggest low awareness and limited utilization of available TH and TM services. Determinants affecting TH and TM included pandemic-based, socioeconomic, cultural, financial, health literacy, and infrastructure-based factors, and those relating to physician implementation. Disparities in utilization of these services were evident among individuals with low digital literacy, older individuals and low-income workers. **Conclusions**: TH and TM are observed to increase the availability and accessibility of preventative and treatment-based health services, especially in regions facing geographic limitations. The findings exhibit the disparities in institution and uptake of these services due to socioeconomic, geographic and cultural aspects. Interventions at the system level, supported by evidence such as multilingual digital platforms, investments in digital infrastructure and coverage in insurance, need to be implemented to ensure equitable utilization of these services in the MENA region.

## 1. Introduction

Telehealth (TH) is a proven mode of health service that can improve the delivery and mobility of healthcare services within different levels of the healthcare system [1]. Telemedicine (TM) is defined as using electronic data and communication technology to create and support health service delivery when geographical limitations pose a barrier [2]. TH and TM are measures that can reduce the cost and lack of accessible healthcare for the general population. These tools can improve the health outcomes of individuals globally.

### 1.1. Telehealth

The use of TH has steadily increased in the healthcare system. Society’s development can be attributed to the advanced and accessible healthcare the population receives. Progress in telecommunication technologies has further highlighted the importance of access to healthcare. This, in turn, influences the various medical and healthcare delivery methods, creating a product called TH [1].

Healthcare systems at the national level are now under pressure to create platforms that can improve the delivery of prompt, accessible, and high-quality healthcare through affordable measures. TH can be a solution here by connecting providers and receivers in remote areas. Structured communication between patients and family caregivers is supported by TH, thereby increasing the flow of clinical knowledge and improving self-management practices. Workforce retention is facilitated by TH in remote areas as it provides opportunity for physicians to maintain communication with peers and continue their professional educational credentials [1].

Most evidence investigating the use of TH and its impact on saving cost, workforce capacity building and other benefits comes from high-income countries in the MENA region, unconsciously creating a bias by not incorporating evidence from low-income countries regarding its specific set of challenges in the adoption of TH. Data and information from LMIC (Lower-Middle Incomes Countries) is sparse but growing. In countries with conflict, such as Afghanistan, Libya, Gaza, Syria, Iraq and Yemen, the majority of telemedicine interventions were created by humanitarian or academic institutions instead of the governmental health systems, with focus on specialist consultation and tele-mental healthcare rather than on primary care services [3].

### 1.2. Telemedicine

For a long time, healthcare workers, physicians and policymakers have been looking for a better alternative to physical consultations. One such innovation is the emergence of TM, which is a compilation of mainstream technology and telecommunication improvements. TM is not new; it uses the telephone for medical consultations or follow-ups between physicians and patients. The radio is often used in military sites and other remote areas to link emergency medical personnel to medical centers. There have been experimental ideas in the field of TM as well, such as when a surgeon performs telesurgery to receive visual and touch-based information to guide and manipulate incredibly precise instruments to perform surgery at a site far away. Between these two aspects of TM lie the variety of video, audio and data transmission technologies and applications. It is often expensive to get equipment for these video conferencing facilities that will ensure great quality and help physicians to watch, hear, examine, question, and counsel patients who are far away so that real-time treatment, follow up and diagnostic feedback can be given. The combination of telecommunications and computer technologies has resulted in the creation of TM to improve healthcare [4].

There is a lack of scientific evidence regarding the attitudes and factors influencing the usage of TH and TM services globally and in the Middle East, making it difficult for public health professionals to monitor and evaluate any progress in this field. Hence, this study aims to address this gap by exploring the attitudes and factors affecting the utilization of TH and TM among individuals. The findings of this review will inform health authorities on the current uptake of TH and TM and its barriers.

## 2. Materials and Methods

The Scopus and PubMed databases were used to compile scientific articles that were aligned to the objectives and were published between 1 January 2020 and 30 June 2025. Although this study is a narrative review, a PRISMA framework was used in order to allow a structured process for creating and presenting the search strategy, which is consistent with recommendations for narrative reviews that seek to improve methodological rigor. The Boolean search strategy used Boolean operators “AND” and “OR” with key terms including “Telehealth”, “Telemedicine”, “Attitude”, “Factors”, “Barriers”, “MENA”, “Middle East” and “Utilization” within selected databases. The Supplementary Material presents the search strings and records identified in each database.

The records were identified if they discussed attitudes, utilization or factors involving TH and TM by screening the titles and abstracts. Shortlisted records were selected if they were peer reviewed articles from official sources such as the WHO, were published between 2020 and 2025 and were in the English language. Records not meeting these requirements were excluded in the final set of records used in the creation of the narrative review. The inclusion criteria may hinder the coverage of evidence and is acknowledged as a limitation in Section 4.

The review was created with importance given to articles with relevant study designs that met the study objectives. To maintain the standard of scientific evidence, peer-reviewed studies were utilized whenever possible. The scientific literature collected was structured around various determinants that affect the use of TH and TM, including attitudes, enablers, barriers and health outcomes relating to TH and TM. Thematic categories were used to group the study’s findings, primarily through rates of uptake and adoption, attitudes among providers and patients and other factors influencing TH and TM. The studies included in the review mostly cover the MENA region.

### 2.1. Operational Definition and Taxonomy

This study defines Telehealth (TH) as the broader term consisting of the remote delivery of health services, clinical education and sharing of information through different telecommunications technologies. Telemedicine (TM) is specifically defined as the delivery of clinical services remotely by a provider or physician. TM falls under TH, and both terms are discussed as reported in the primary literature sources. Teledentistry, telepharmacy and telenursing have been considered to be sub-domains under TH.

### 2.2. Risk of Bias Assessment

The AXIS tool [5] was used to perform the risk of bias assessment among the cross-sectional studies included in the review (Table 1). Ten studies included in the review used a cross-sectional study design.

## 3. Results

Three thematic categories were used to provide structure for the review: (1) Utilization characteristics of TH and TM. (2) Attitudes towards TH and TM by providers and patients. (3) Determinants affecting TH and TM uptake. The scientific evidence focuses on the MENA region with selected inclusion of global studies to improve the context.

### 3.1. Utilization

#### 3.1.1. Telehealth

COVID-19 brought to light the importance of more sustainable healthcare delivery services in the global economy. It was at this time that several governments and institutions of importance realized the extent to which TH can help the community in global emergencies and the way we respond to them. The lockdown measures taken during COVID-19 enhanced the need for TH as a platform to help those who thought they might be infected. TH also seemed to be the best measure for providing remote assessment or triage and the delivery of treatment and care. TH also acted as a convenient and accessible way to receive care and treatment for people with infections without being in high-risk exposure-based environments like hospitals and waiting rooms in medical centers. TH has been used to handle emergency situations within the MENA region, especially for care in conflict-affected areas [3,6,16].

In examining the use and patient acceptability of TH before and after the pandemic, it was observed that prior to the COVID-19 pandemic, 88.6% of participants from the Middle East and North Africa (MENA) region had no prior exposure to TH. After the pandemic, TH use has increased by 251%. Most TH users (89%) used TH through virtual visits with specialists [7]. Several reasons could be attributed to the increase in the use of TH in the MENA region, one of which is the broader scale of implementation of TH services to reduce the risk of infection by physically going to hospitals and clinics. Another reason is the issuance of lockdowns, which made patients seek forms of care that were safer and more convenient during the COVID-19 pandemic. Overall, 66% of participants in a study revealed that TH made them feel safer and protected from infection during the pandemic [7].

**Figure 1:**
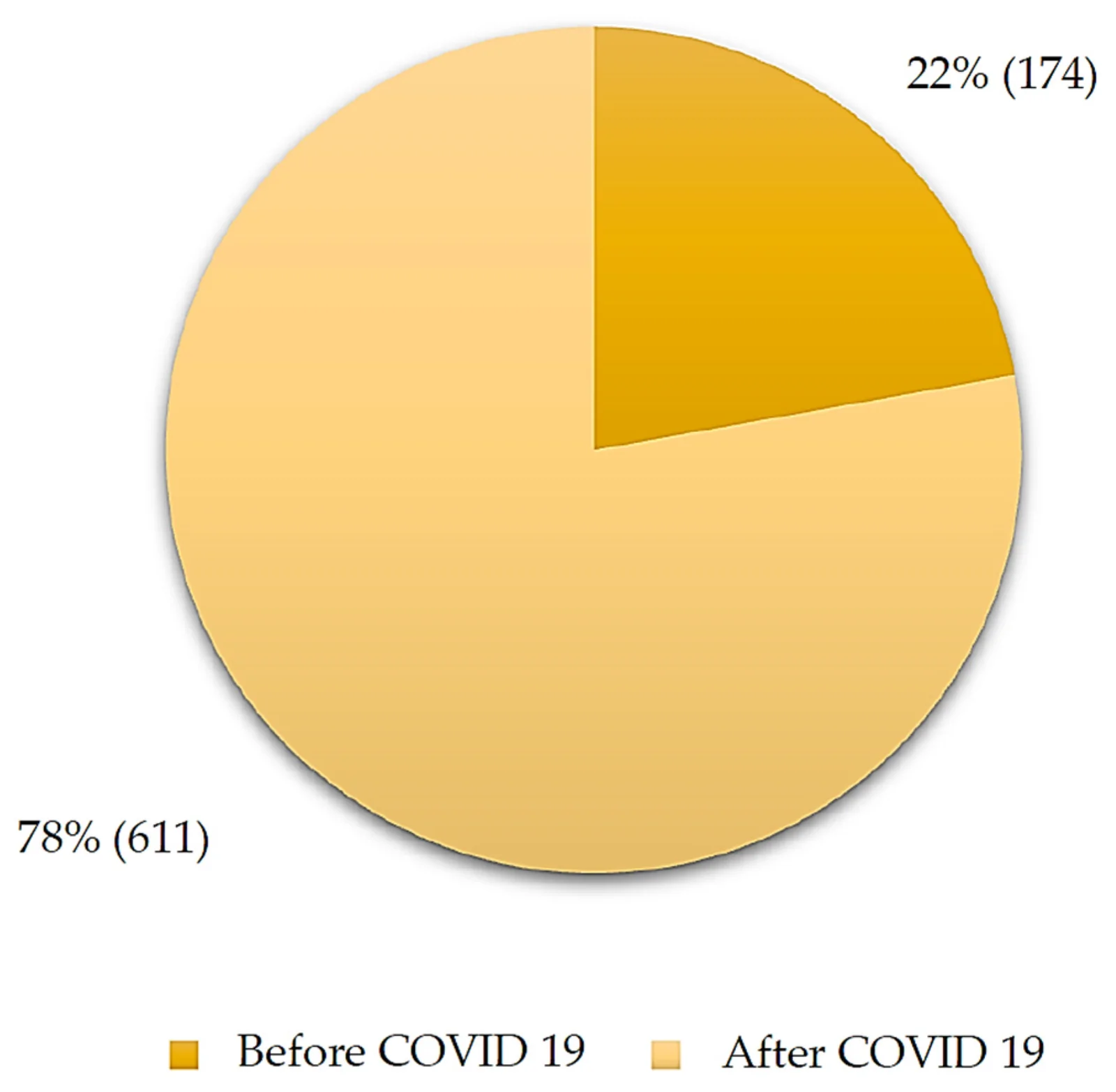
Utilization of Telehealth before and after COVID-19 in the MENA region [7].

Similar to the rest of the world, the pandemic increased the use of TH in the outpatient setting in the MENA region. The healthcare authority in Dubai, also known as the Dubai Health Authority (DHA), made sure to provide healthcare service provision to the public during the pandemic. DHA issued the first clinical guidelines regarding TH use in 2017, with revisions made in 2021 [8]. The DHA guidelines cover six key areas, including Teleconsultation, Telediagnosis, Telemonitoring (or remote patient monitoring), Mhealth (or mobile health), telerobotics and robot-assisted services, and tele-pharmacy. The purpose of the standards was to establish minimum requirements for providing safe, efficient and high-quality TH services, to encourage innovation in healthcare and health technology and to create guidelines for monitoring and evaluating TH services provided by DHA facilities [17]. A cross-sectional study observed the rates of TH use among patients from 2020 to 2021. Of the total number of scheduled TH visits, 78.55% of them were completed. A shorter turnaround time in receiving appointments was noticed for older patients, patients who had visits in 2020, non-Emiratis, patients having video visits and patients who had family medicine appointments [8] (Figure 1).

#### 3.1.2. Telemedicine

Due to the COVID-19 pandemic in 2020, it became important that healthcare services were delivered remotely using TM [18]. Technology has helped pave the way for remote consultations, monitoring, and patient education via phone and videoconferencing during the pandemic [19]. TM enables patient care while also reducing the need for face-to-face interactions, which significantly lowers the spread of infection and the requirement for beds at healthcare facilities. TM can be used for the current management of chronic diseases, adherence to medicines, medical consultations and other online services [20]. In terms of the adoption of TM in the global arena and its benefits, the Italian Society of Cardiology used TM for cardiovascular disease patients in the prehospital triage for ST-elevated myocardial infarction (STEMI) cases and in the case of remote monitoring by primary care physicians [21]. In pediatric cardiology, the American Heart Association (AHA) stresses the importance of advanced video technologies like tele-echocardiography, prenatal diagnosis uses fetal echocardiography, echo tests for confirmation of a diagnosis, external rhythm monitoring, personal tele-electrophysiology and for screening of congenital heart diseases [22]. TRANSIT-stroke is a program created in Germany where a TM network was built for rural hospitals. This led to better patient outcomes as neurological assessments were done quicker, the treatments were given out within the optimal timeframe and most importantly, there was 24 h access to a neurologist [23].

In the Middle East, TM has the potential to improve the accessibility of various general and specialist health services for residents. The MENA region has a fast-growing and diverse population, which has led to differences in the distribution of healthcare services among all areas of each country. A prominent challenge in this region is the lack of healthcare in rural and lower socioeconomic areas. Most hospitals and clinics are situated in major cities in these countries. Transportation to and from these healthcare centers acts as a challenge for vulnerable or disadvantaged subgroups of the population in this case [24]. The implementation of TM is slow because of the diversity and complexity of the cultural and social backgrounds of the individuals in this region. TM programs were introduced in the 1990s in countries such as Saudi Arabia, Bahrain, Iraq, Syria, Qatar, Jordan, Kuwait, Turkey, Iran and the UAE, but they have not been used effectively. Most of these TM programs did not progress as per expected targets [25].

One example of TM in the MENA region is the virtual clinical consultation called “Doctor for Every Citizen”. The smart government application facilitated TM and helped Dubai connect patients to world-class physicians no matter the distance [9,26].

In 2019, COVID-19 emerged in Wuhan, China. The severe acute respiratory syndrome, which was due to the SARS-CoV-2 pathogen, was then transmitted to over 100 countries. Due to the pandemic, many countries have started using TM to provide care without putting patients at risk by coming to the healthcare center [9]. A study that looked at the utilization of TM during the pandemic observed that 31.3% of participants used TM during the 2019 pandemic. There are many reasons for the lack of TM usage. The first reason was that people were not aware of the existence of TM (38.3%). The second major reason was that people did not possess the knowledge of how to use TM (33.5%). Additionally, 89.7% of TM users opted for tele-pharmacy, 78.2% of TM users used it for teleconsultation, and 23.0% of users used it for telediagnosis during the COVID-19 pandemic; 94.6% of TM users also reported that they sought TM to ask a pharmacist’s advice about instructions for taking medications, whereas 85.1% used TM to order nonprescription drugs and 80.8% used it to obtain physician advice [10]. Perceived usefulness, or PU, and behavioral intention of use, or BIU, were seen as critical elements when it comes to the use of TM services. Furthermore, the behavioral intention of use was also seen to be a predictor of the actual usage of TM [11].

The COVID-19 pandemic contributed to a significant uptake of TH and TM across the world. Nevertheless, there was variation in its adoption, implementation and progress among different groups of individuals. Countries such as Singapore, with higher gross domestic product, had demonstrated an innovative and efficient use of TH and TM to improve triage, access to specialists and sustainable care, whereas the utilization of TH and TM is not uniform throughout the MENA region. Despite regulations promoting TH and TM, and the existence of governmental initiatives and policy frameworks, the utilization of these digital services is limited by a lack of perceived effectiveness, low digital literacy and low awareness.

The contradiction between evidence of consistently high positive attitudes towards these services along with the low rates of utilization in the MENA region is to be interpreted carefully. Favorable attitudes do not often translate into action, which in this case refers to the action of using TH and TM when the services are available. This is due to many barriers at different levels, including structural barriers and individual barriers. Structural limitations can include lack of insurance or limited insurance coverage, low digital infrastructure and poor literacy about digital health. This differentiation is significant to policy makers and authorities as it facilitates the right course of strategy to increase uptake of digital services rather than relying only on awareness campaigns to address individualistic barriers. Awareness campaigns and health promotion alone cannot improve the utilization of digital services if systematic barriers are not addressed simultaneously. High income countries such as Australia and Singapore present evidence on how improving infrastructure and training measures lead to positive attitudes and utilization, hinting that the possible gap in uptake for digital services in the MENA region is substantially due to structural factors rather than attitudinal determinants (Table 2).

### 3.2. Attitudes Toward TM and TH

#### 3.2.1. Telehealth

**Figure 2:**
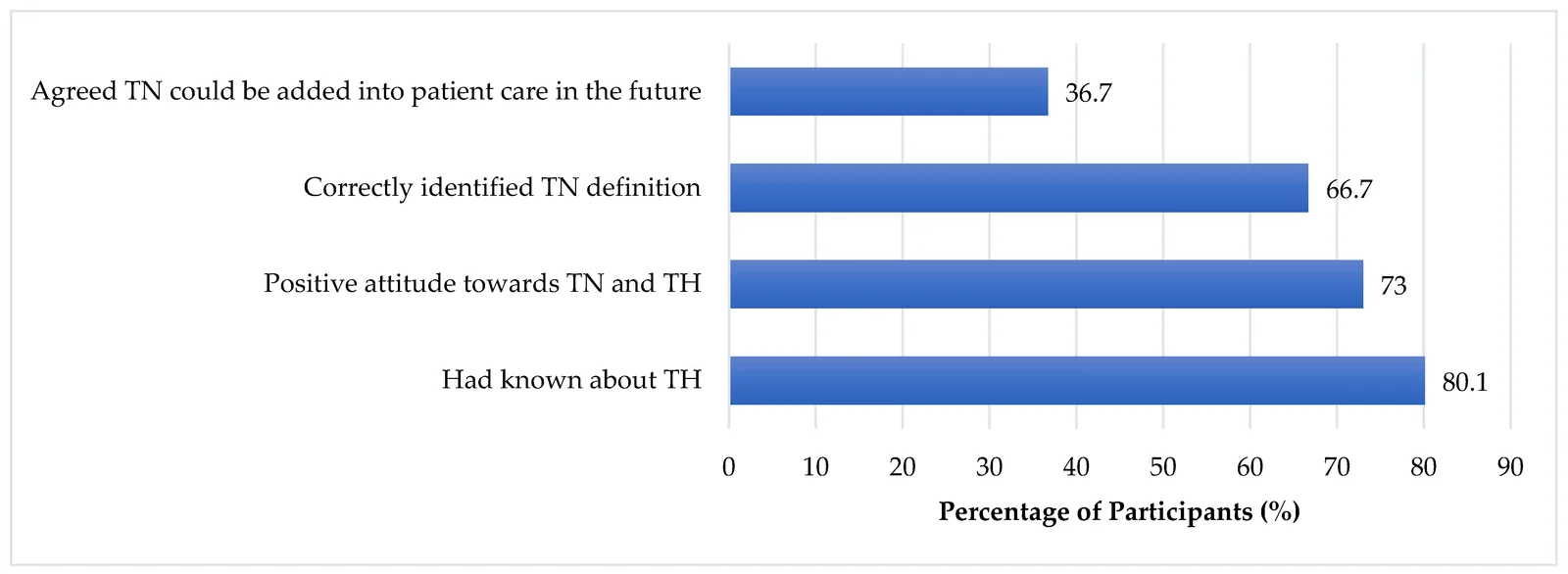
Awareness and attitudes about telenursing and telehealth by nurses and midwives in Iran [12]. Note: TN—telenursing, TH—telehealth.

Attitudes towards TH and TM can influence the uptake of these services by patients and the delivery of these services by providers. A study in Iran examined the attitudes and awareness of Iranian clinical nurses and midwives towards telenursing and TH. Overall, 66.7% of participants identified the definition of telenursing, whereas 80.1% of participants knew about TH. Additionally, 73.0% of participants had a positive attitude towards both telenursing and TH. Another interesting finding was that midwives presented a more frequent positive attitude towards telenursing and TH than nurses. Almost one third (36.7%) of participants agreed that telenursing could be integrated into patient care in the future [12] (Figure 2).

#### 3.2.2. Telemedicine

To increase the advantages of TM, it is important that we know the attitudes of healthcare professionals towards its application. A global survey done among healthcare professionals revealed that 90.9% had heard about TM, 65.3% had witnessed TM, and 74.6% were aware of how TM would be used in practice. Additionally, 72.2% were aware of different tools used for this specific technology. Positive attitudes were generally noted when providing healthcare remotely. Furthermore, 80.6% agreed that it would reduce transportation costs, 80% agreed that it would reduce staff workload, and 83% believed that it would save the physicians time. On the other hand, 40.5% of healthcare professionals thought that TM would risk the confidentiality and privacy of patient information, and 20% of respondents agreed that it would increase staff workload [28].

**Figure 3:**
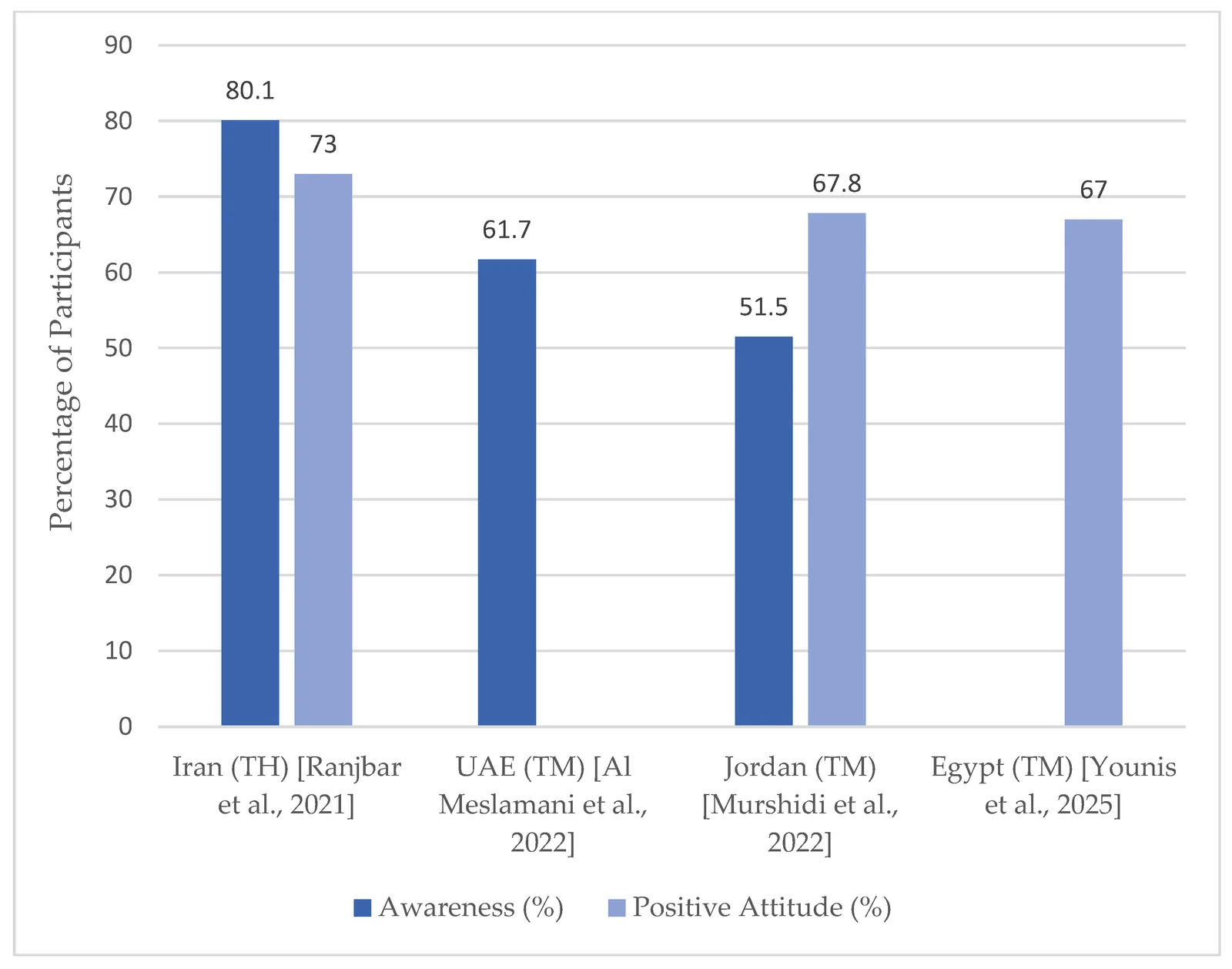
Awareness and positive attitudes towards TH and TM [10,12,13,14].

The scientific literature presents an overall positive attitude towards TH and TM among patients and healthcare providers. Positive attitudes were mainly due to the convenience, efficiency and affordability offered by TH and TM services. Despite these advantages, some concerns were stated with respect to effectiveness, privacy and inability to perform physical examinations, which contributed to less favorable attitudes towards the services. The practicality of integrating TH and TM and the possibility of these services to increase the workload of physicians and lower the rapport between physicians and patients are other contributing factors for negative attitudes towards TH and TM. This implies that positive attitudes and better integration measures need to be taken to ensure sustainable implementation of TH and TM in the healthcare system (Figure 3).

### 3.3. Factors Affecting the Use of TH and TM

#### 3.3.1. Pandemic-Based Factors

During the COVID-19 pandemic, scientists from many countries looked into the possibility of using TM-based technologies and reevaluated their strengths, weaknesses, and suitability for modern electronic means used in healthcare systems. TM has already been proven to be effective in different aspects of healthcare, including for the treatment of non-communicable diseases such as diabetes mellitus and cardiovascular diseases, by promoting high-quality treatment from a distance. The prominent advantages of TM include its ability to save time and manpower and ease of contactless healthcare service delivery, especially during outbreaks of communicable diseases [29]. The use of TH increased drastically during the COVID-19 period for providing healthcare service delivery [8].

Although the COVID-19 pandemic led to an expansion of available TH and TM services, the uptake among vulnerable groups such as low-income workers remained uneven [30,31,32]. Failure to reach these subgroups can lead to public health issues through increasing the risk of undetected transmission of infection diseases, greater burden on emergency services and impact on the economy due to absences and illness among the workforce. Additional structural challenges of TH and TM technology such as lack of full coverage in insurance, barriers due to language and comprehension abilities, and differential employment environment factors can influence access to these services.

#### 3.3.2. Cultural Factors

Providing healthcare services while taking into account the cultural and religious characteristics of the population helps to effectively deliver healthcare services while improving relationships with patients and gaining their trust. Studies suggest that sensitive cultural issues impact individuals’ perceptions and attitudes to TM in countries such as Saudi Arabia, Iran, Egypt, Kuwait, Iraq, Yemen and Syria. Cultural factors impacting TH and TM use can include religious and social restrictions, resistance to change, linguistic distinction and traditional beliefs. In the case of female clinical staff, some individuals prefer not to be recorded or filmed. Fear of media-based data getting stolen, leaked, or lost by unauthorized people is a major reason that women cannot provide and use these services [25].

In comparison to the general population, there are several cultural and religious factors that can affect the use of TH and TM. Compared to men, women have greater hesitancy to use TH and TM services due to the nature of delivery, which includes video and electronic media. Other cultural and religious groups may place less belief in the effectiveness of TH and TM and prefer face-to-face interactions with their doctor, which decreases their use of TH and TM compared to the general population.

#### 3.3.3. Socioeconomic Factors

Studies suggest a positive attitude and general acceptance towards TH services among the population in the MENA region. One study by Alnakhi et al. identified that users’ socioeconomic characteristics affected the utilization of TH services. They found that women and the younger demographic were more likely to consume TH services, which is consistent with other international findings [8]. Other studies suggest that patients who are older have a higher likelihood of using TH than visiting primary healthcare centers as it requires less movement and improves their safety by preventing falls and other health hazards. TH could also be used by elderly patients from the comfort of their own homes [33].

#### 3.3.4. Literacy Factors

A common reason for the lack of use of TM services during COVID-19 was a lack of knowledge about the availability of TM or how to use it. The most frequently used TM services include tele-pharmacy, teleconsultation, and telediagnosis. Overall, 38.3% of participants in a study by Al Meslamani et al. stated that they were unaware of the concept of TM and where to access it. Additionally, 33.5% of these participants were unsure of how to use TM services and how they can be helpful. Furthermore, 23.8% of participants were not able to trust the effectiveness of TM from a clinical perspective [10]. Other barriers that can play a role are the negative effects of available digital health literacy, which is inaccurate but easily accessible through mobile phones due to the internet [29].

Among various vulnerable subgroups, it is evident that digital health literacy impacts the use of TH and TM. Although there are several available TM services in the community, there are gaps in the uptake of digital health services due to low awareness, low perceived effectiveness and privacy concerns.

#### 3.3.5. Physician Implementation Factors

A systematic review identified several reasons for introducing TM in lower and middle-income countries. The reasons are to maintain non-emergency healthcare, improve access to health providers and reduce the risk of infection among health users and providers during the COVID-19 pandemic. Overall, healthcare providers and users have shown a high level of acceptance towards TM services [33]. Another study by Hilty et al. identified several barriers to physicians’ adoption of TH. These barriers include clinical barriers and workforce and technology barriers. Clinician barriers include concerns about the difficulty in establishing a rapport or a good clinical relationship with patients through TH. Many physicians and patients prefer face-to-face consultations that enable both parties to understand key verbal and non-verbal signs and communicate effectively. The best strategy for overcoming this barrier is providing technological training for clinicians. The next significant barrier is related to workflow and technology, wherein physicians and healthcare staff need additional time to plan, organize and coordinate operations for a TH-based visit, which is not required in face-to-face encounters [34].

The method of TH and TM implementation by physicians will impact the use of these services in different health systems, particularly in low- and middle-income countries. By addressing barriers related to patient interaction, integration into clinical practice and the ability to use technology, physicians can improve the delivery of TH and TM to ensure its sustainable use and acceptance in the MENA region.

#### 3.3.6. Financial Factors

Research still does not provide clarity on the cost of implementation of TM. The cost can vary depending on the specific TM tool used, and this provides a sense of uncertainty that prevents investments in this field. The long-term sustainability of health systems worldwide is a significant concern for governments. Withstanding the impact of increasing costs and increasing the ratio of health expenditure to gross domestic product, the financial stability of healthcare systems may fluctuate.

To establish TH and TM at the health system level in the MENA region, researchers need to implement cost-effectiveness studies across primary care and tertiary care centers to facilitate the introduction of national TH and TM services. TH and TM will help the governments to reduce costs and long-term expenses for physical infrastructure and improve affordable health services for the community. However, as the cost savings from these digital services are not straightforward and automatic, the evidence suggests that TH can reduce costs in the long run through reducing costs and loss of productivity incurred while commuting [35].

#### 3.3.7. Accessibility- and Infrastructure-Related Factors

The utilization of TM was found to be affected by other factors, including limited insurance coverage (74.2%), issues with Internet connectivity (53.2%) and delays with responses from medical service centers (28.6%). Many patients preferred interactions with physicians when discussing health issues, and about 23.3% of participants in a study reported that they needed a physical examination that could not be done through TM [10].

Satisfaction with TM was another important factor in deciding the use of TH and TM among individuals. Satisfaction with TM was seen to be negatively correlated with the preference to seek regular health care services. This meant that individuals who regularly sought healthcare services were less likely to be content with TM-based services. Participants who were satisfied with TM services were also found to be more likely to use these services in the future [10].

As these factors accumulate, they shape the usage of TH and TM among the working population. Challenges related to insurance coverage, variable internet coverage, logistical issues and policies concerning these digital services can influence the frequency of utilization of TH and TM. The satisfaction and perceived effectiveness of TH and TM by individuals will determine if and how many times people will look to these services for their health needs. Facing these challenges will require action from system-level interventions to community-based health promotion. Examples include the development of multilingual digital interfaces, increasing accessibility to the internet in areas with lower SES and creating stations with personnel that can help individuals with lower digital literacy to use these services. Table 3 presents key findings about various enablers and barriers affecting the use of TH and TM. 

## 4. Discussion

### 4.1. Understanding TH and TM Adoption Through TAM and UTAUT Frameworks

The Technology Acceptance Model (TAM) and the Unified Theory of Acceptance and Use of Technology (UTAUT) are excellent frameworks validated to critically analyze the adoption of remote healthcare services [38,39]. The TAM framework explains the adoption of digital services through perceived usefulness and the perceived ease of use, while the UTAUT framework looks at performance expectancy, social influence, effort expectancy, and facilitating conditions that can become predictors for behavioral intention. When applied to evidence from the MENA region, Abdool et al. found that behavioral intention of use was a high and significant predictor for uptake of TM, which aligns with the reasoning of the TAM [11].

The UTAUT model and the construct of performance expectancy is supported by the scientific literature. A study by Naqvi et al. found that 83% of health care professionals thought that TM can save physicians time, and 80% of participants believed it can reduce staff workload [28]. Several studies also contradict this evidence, citing that healthcare professionals felt that there would be difficulties in having clinical rapport and communication with patients and it would cause additional coordination work in healthcare facilities [34]. The opposing views regarding the benefits of digital services among healthcare professionals will need to be investigated on a deeper scale to understand which professions face the most difficulty and which tasks can be handled with more ease. The transition of most hospitals from physical patient files to digital files was once opposed but is now the norm, and similarly digital services such as TH and TM have the potential to cover a lot of clinical tasks in a more time-efficient manner with benefits to both the patient and the healthcare professional involved.

However, the discrepancy happens when considering the facilitating conditions in place, such as the awareness of available services among individuals, digital infrastructure and user support in countries in the MENA region. Al Meslamani et al. found that 38.3% of TM non-users were unaware that such services existed and 33.5% of individuals did not know how to access the platforms [10]. In the UTAUT context, cultural norms around video recording by female clinicians and privacy concerns conveyed by 40.5% of healthcare professionals contribute to the negative social influence signals [28]. Using evidence from the MENA region, the frameworks reveal that TH and TM adoption is influenced not only by motivational deficits but due to structural shortcomings, which is an appropriate distinction for policy makers to consider when prioritizing development of digital services.

### 4.2. The Gap Between Attitude and Uptake of TH/TM Services

The most important finding obtained in this review is the gap found between preferable attitudes towards TH and TM and low behaviors relating to utilizing them. Shamiyah et al. observed that although 66% of TH users had felt safer using TH during the pandemic, only 36.8% of users hoped to continue after the pandemic [7]. This is further supported using the TAM and UTAUT theories, which explain that the adoption of digital services can be sustainable only when facilitating conditions can promote positive attitudes to positive behavior.

### 4.3. Inverted Barrier Structures in the MENA Region

When comparing TH and TM utilization trends within the MENA region in comparison to global evidence, they seem to be opposed. In a global context, in high-income countries such as Australia and Singapore, patients are highly accepting of the digital services and the barriers mainly lie from the perspective of physicians who are concerned about losing rapport with patients, disruptions in work and liability for institutions [33,36,40]. However in the MENA region, the structure for determinants are inverted, where the patients are less aware of where to access TH and TM and they are less aware of the availability of services, but the infrastructure and regulatory policies are advanced [10,37]. It is vital to take this inversion into account, as it provides guidance on the priority reforms that countries in the MENA region must focus on. Reforms should focus on policy that would improve access to information about TH and TM among individuals and how to access it.

### 4.4. Implications for Health Policy and Structural Factors

A significant structural factor to consider is the limited coverage for TM by insurance. About 74.2% non-users state insurance as the main barrier when accessing TM. This barrier is not an individual determinant, but remains a structural barrier that can only be counteracted through structural changes in the expansion of digital services covered by insurance. When governments can lead this reform, it will help individuals not face any financial burdens to use TM and TH. Additionally, other structural barriers such as inconsistent access to internet connectivity and concerns with physician workflows need to be addressed to remove uptake [10].

Governments may be hesitant to move towards investing in these digital services and their related infrastructure due to the rising health expenditure within countries and the evidence on guaranteed returns. Cost effectiveness analysis needs to be conducted in countries of the MENA region to support the adoption of TH and TM.

### 4.5. Limitations

This review has some limitations. It could potentially have been influenced by selection bias due to the study design being a narrative review. As the review only incorporated sources in English, evidence written in Arabic or other local languages was not interpreted in this review. No risk of bias assessment was performed in this review, which can limit causal inference due to the collection of different study types, including cross sectional and other observational study designs. The TAM and UTAUT frameworks were utilized retrospectively to critically analyze the studies included in the review; the authors suggest future studies that focus on a specific model and its application in digital health services.

### 4.6. Future Directions

Future research must focus on longitudinal studies that analyze the adoption of digital services across the pandemic and the implementation of cultural and linguistically adapted models in the MENA region. Evidence on cost effectiveness analysis across various geographical settings in the MENA region and the adoption rates of digital services among vulnerable groups such as the elderly, unskilled workers, children, and pregnant women must also be investigated.

## 5. Conclusions

This narrative review finds that, despite the high rates of positive attitudes found across the MENA region, there exist low and inconsistent rates of utilization of TH and TM. This gap from attitude to utilization can be attributed to structural barriers through most countries in the MENA region. Although the pandemic drove an increase in the awareness and distribution of these digital services, it was short-lived and promoted due to necessity in the period and mainly focused on areas with higher SES and literacy. In terms of health policy, it is imperative that system-level standards and reforms are used in addition to awareness campaigns and health promotions. Reforms must work on insurance coverage, digital infrastructure, linguistically and culturally adapted models, and easy user interfaces to ensure equitable adoption of TH and TM in the region. The transition between traditional diagnosis and treatment may benefit from the inclusion of these digital services to save time and cost.

## Figures and Tables

**Table 1:** Risk of assessment using the AXIS Tool for cross sectional studies.

Risk of Bias	Mahgoub et al. [6]	Shamiyah et al. [7]	Alnakhi et al. [8]	Swidan et al. [9]	Al Mesl et al. [10]	Abdool et al. [11]	Ranjbar et al. [12]	Murshidi et al. [13]	Younis et al. [14]	Kamal et al. [15]
Introduction										
Were the aims/objectives of the study clear?	Y	Y	Y	Y	Y	Y	Y	Y	Y	Y
Methods										
Was the study design appropriate for the stated aim(s)?	Y	Y	Y	Y	Y	Y	Y	Y	Y	Y
Was the sample size justified?	Y	N	Y	Y	Y	Y	Y	Y	Y	Y
Was the target/reference population clearly defined? (Is it clear who the research was about?)	Y	Y	Y	Y	Y	Y	Y	Y	Y	Y
Was the sample frame taken from an appropriate population base so that it closely represented the target/reference population under investigation?	N	N	Y	Y	N	Y	N	Y	N	Y
Was the selection process likely to select subjects/participants that were representative of the target/reference population under investigation?	N	N	NA	NA	N	N	N	N	N	N
Were measures undertaken to address and categorize non-responders?	N	N	NA	NA	N	N	N	N	N	N
Were the risk factor and outcome variables measured appropriate to the aims of the study?	Y	Y	Y	Y	Y	Y	Y	Y	Y	Y
Were the risk factor and outcome variables measured correctly using instruments/measurements that had been trialled, piloted or published previously?	Y	N	NA	NA	Y	Y	Y	Y	Y	N
Is it clear what was used to determined statistical significance and/or precision estimates? (e.g., *p* values, CIs)	Y	Y	NA	NA	Y	Y	Y	Y	Y	Y
Were the methods (including statistical methods) sufficiently described to enable them to be repeated?	Y	Y	Y	Y	Y	Y	Y	Y	Y	Y
Results										
Were the basic data adequately described?	Y	Y	Y	Y	Y	Y	Y	Y	Y	Y
Does the response rate raise concerns about non-response bias?	N	Y	NA	NA	N	N	N	Y	N	Y
If appropriate, was information about non-responders described?	N	N	NA	NA	N	Y	N	N	N	N
Were the results internally consistent?	Y	Y	Y	Y	Y	Y	Y	Y	Y	Y
Were the results for the analyses described in the methods presented?	Y	Y	Y	Y	Y	Y	Y	Y	Y	Y
Discussion	Y	Y	Y	Y	Y	Y	Y	Y	Y	Y
Were the authors’ discussions and conclusions justified by the results?	Y	Y	Y	Y	Y	Y	Y	Y	Y	Y
Were the limitations of the study discussed?	Y	Y	Y	Y	Y	Y	Y	Y	Y	Y
Other										
Were there any funding sources or conflicts of interest that may affect the authors’ interpretation of the results?	Y	Y	Y	Y	Y	Y	Y	Y	Y	Y
Was ethical approval or consent of participants attained?	Y	Y	Y	Y	Y	Y	Y	Y	Y	Y

Y—Yes, N—No, NA—Not Applicable.

**Table 2:** Attitudes and determinants affecting telehealth and telemedicine use in the MENA region.

Author and Year	Country	Target Population	Key Findings
Beheshti et al., 2022 [1]	Iran	Primary health care	Despite steady increase in TH, it is limited within primary care centers and facilities. Determinants include reluctance by physicians, cultural and health policy barriers.
Parkes et al., 2022 [3]	Afghanistan, Libya, Syria, Yemen, Iraq, Gaza	Conflict-Affected Countries	Majority of TM interventions are started through humanitarian organizations or academic institutions and not national bodies. Focused on telemental health and teleconsultation without inclusion of primary care.
Eljack et al., 2023 [16]	Sudan	Physicians	In conflict-based settings, physicians used teleconsultation through voice calls to provide possible care; emphasis on the requirement of national protocols that are standardized.
Mahgoub & Nouri, 2025 [6]	Sudan	600 internally displaced persons (IDPs) in Shendi City	Reported 77.5% ownership of smartphones and 73% had access to internet among urban participants. In 2022, approximately 29% national internet penetration observed, indicating possible increase in barriers faced by rural participants.
Shamiyah et al., 2023 [7]	MENA (GCC)	1524 respondents through online survey	Prior to the pandemic, 88.6% had no exposure to TH; use of TH increased by 251% after pandemic. Of active users, 89% used the virtual specialist visits; majority had positive attitudes; 66% participants felt safer with TH services
Alnakhi et al., 2023 [8]	UAE	241,822 appointment records for TH	78.55% of TH appointments completed; non-Emiratis, older patients and video visits found to have shorter turnaround times. DHA covers six TH service-based areas.
Abouzid et al., 2022 [24]	MENA	Challenges and benefits in MENA Region	Accessibility improved due to TM in MENA region; disparities found in rural and low SES areas. Higher concentration of hospitals in cities. Slow implementation of TM due to cultural and social factors.
Al-Samarraie et al., 2020 [25]	Middle East	43 articles from 2010 to 2020	Inadequate progress with expansion of TM in Middle East due to economical, cultural, technological, legal and individual barriers. Low digital literacy identified.
Swidan et al., 2022 [9]	UAE	Patients who used DHA TM services (2019–2021)	38.3% did not know about TM. 31.3% utilized TM during COVID-19 pandemic; 33.5% were unsure of how to use TM; 89.7% used TM for tele-pharmacy. COVID-19 increased uptake of TM.
Al Meslamani et al., 2022 [10]	UAE	General Population	Predictors of TM found included PU and BIU. Limited insurance (74.2%); internet difficulties (53.2%); delays in responses (28.6%).
Abdool et al., 2021 [11]	UAE	General Population	PU and BIU are significant in prediction of TM use.
Ranjbar et al., 2021 [12]	Iran	Nurses and Midwives	TH was known about by 80.1%. Positive attitudes (73.0%) with midwives observed to be more positive than nurses; 36.7% of participants encourage integration of telenursing in future.
Ali et al., 2025 [27]	Gaza, West Bank	20 healthcare volunteer workers	The WhatsApp application was used to communicate in spite of connectivity issues and power outrages. In Gaza, EMR was mainly replaced with paper records and phone photos. The fall of adequate infrastructure influenced digital tool use mostly rather than attitudes with limited EMR access forcing medical students to become scribes.
Murshidi et al., 2022 [13]	Jordan	1201 participants from online surveys	67.8% of participants were willing to use TM. Perceived ability was a predictor of positive attitudes, with those living in southern Jordan predicted to have poorer attitudes.
Younis et al., 2025 [14]	Egypt	1000 Tanta University Hospital physicians and staff	38.2% of staff and 50% of physicians reported using TM, with two thirds of participants having good knowledge or attitude. Major barrier was limited technical infrastructure.
Kamal et al., 2024 [15]	Egypt	214 Physicians who used national e consultation tool	97,000 consultations performed in approximately 3 years, mostly for pediatrics and dermatology specialties; majority of physicians stated high cost savings for patients with limitations in implementation due to acceptance and technical barriers.

SES—Socioeconomic status, PU—perceived usefulness, BIU—behavioral intention of use, MENA—Middle East and North Africa.

**Table 3:** Key findings regarding enablers and barriers for utilization of TH and TM.

SR No.	Factors		Key Findings	Article References
1	Pandemic-Related	Enablers	Rapid increase in TH and TM adoption due to COVID-19; post pandemic use increased by 251% in MENA; contributed to triage, monitoring remotely, remote treatment and care; improved coverage for TH by DHA.	[7,8,17,18,19,20,36]
		Barriers	Vulnerable subgroups like unskilled workers affected more than the general population during this period; disproportionality excluded from the expansion of TH and TM.	[30,31,32]
2	Cultural	Enablers	Positive attitudes reported for teledentistry in Indonesia, for telenursing by nurses and midwives in Iran, for TH and TM by healthcare professionals globally; good levels of awareness and readiness reported by healthcare professionals.	[12,28,37]
		Barriers	Restrictions or stigma for video recordings due to religious or cultural reasons, especially by female clinicians; fear of leak of confidential information; high preference for onsite consultations by patients and physicians; natural resistance to change traditional treatment methods and beliefs.	[23]
3	Socioeconomic	Enablers	People of female gender and young age seen to be more likely to use TH; Older patients can access in-home TH and reduce the need to commute and reduce the risk of falls.	[8,33]
		Barriers	Differences in income and work type/environment can reduce the adoption of these digital services; other socioeconomic disparities can create inequitable use in the MENA region.	[24,25]
4	Literacy	Enablers	Countries with increased digital literacy present sustainable TH integration and good outcome; clinician hesitancy and other barriers are removed through technology training.	[34,35,36]
		Barriers	23.8% participants were not convinced of the clinical effectiveness; negative impacts of health misinformation; lower digital literacy observed among elderly and low-income groups.	[10,29]
5	Physician Implementation	Enablers	High rates of provider acceptance globally; improvements in accessibility for providers and patients in LMICs due to TM; substantially reduces infection risk; training is a significant determinant; guidelines by governments improve implementation.	[8,17,33]
		Barriers	Challenges in establishing rapport by video; physicians concerned that they cannot understand nonverbal cues through TH and TM; extra time required for coordination to arrange TH visits, barriers based on workforce, reluctance by clinicians seen in countries in the Middle East and globally.	[1,25,34]
6	Financial	Enablers	TH is associated with lower costs due to less commuting and lower requirements for expensive specialist follow up; transport burden is lowered by teleconsultations and tele-pharmacy; high probability of increasing expenditure for patients who do not have insurance.	[9,35]
		Barriers	Lack of or limited insurance; complex budgets for TM tools introduce uncertainty about investments; implementation of TH does not guarantee to lower system costs; unclear ROI in the long term.	[10,35]
7	Accessibility and Infrastructure	Enablers	National frameworks and standards set in place by governments; infrastructure for video visits can increase specialist access; apps released by governments can improve uptake.	[8,9,26,36]
		Barriers	Connectivity and access to internet; 28.6% delays in responses sent by healthcare facilities; gaps in technology in rural areas across MENA region; ethical management of data and privacy concerns.	[9,10,13,22,24,29]

DHA—Dubai Health Authority, ROI—Return of Investment.

## Data Availability

No new data were created or analyzed in this study. Data sharing is not applicable.

## Supplementary Materials

The following supporting information can be downloaded at https://www.mdpi.com/article/10.3390/healthcare14162442/s1, Table S1: Electronic Search Strategy.

## Abbreviations

The following abbreviations are used in this manuscript:

TH	Telehealth
TM	Telemedicine
TD	Teledentistry
UAE	United Arab Emirates
STEMI	ST-elevated myocardial infarction
AHA	American Heart Association
DHA	Dubai Health Authority
MENA	Middle East and North Africa
