# Supplementary material for: Attitudes and Determinants Affecting Telehealth and Telemedicine Use in the MENA Region: A Narrative Review
Source: Healthcare (Basel). 2026 Aug 7;14(16):2442. doi: 10.3390/healthcare14162442 (PMC13512277; doi:10.3390/healthcare14162442)
Supplement: Supplementary file 1 [file healthcare-14-02442-s001.zip › healthcare-4403441-supplementary.pdf]

Supplementary Material

Table S1. Electronic Search Strategy (16th January 2026)

Database	Search No.	Search String	Records Retrieved
Scopus	S1	(Telehealth OR Telemedicine) AND MENA AND Utilization	733
	S2	(Telehealth OR Telemedicine) AND MENA AND Enabler	115
	S3	Telehealth AND MENA AND Attitude	437
	S4	Telemedicine AND MENA AND Attitude	646
	S5	Telehealth AND MENA AND Determinant	399
		Telehealth AND Middle East AND Attitude	1159
		Telehealth AND MENA AND Barrier	581
		Telemedicine AND MENA AND Barrier	828
		Telemedicine AND Middle East AND Attitude	2050
		Telemedicine AND North Africa AND Attitude	1383
		**Total Scopus records that are identified**	**8331**
PubMed	P1	(Telehealth OR Telemedicine) AND MENA AND Utilization	18
	P2	(Telehealth OR Telemedicine) AND MENA AND Enabler	4
	P3	Telehealth AND MENA AND Attitude	6
	P4	Telemedicine AND MENA AND Attitude	6
	P5	Telehealth AND MENA AND Determinant	10
	P6	Telemedicine AND MENA AND Determinant	8
	P7	Telemedicine AND Determinant	23496
	P8	(Telehealth OR Telemedicine) AND Utilization AND Attitude	4631
		Telehealth AND Middle East AND Attitude	287
		Telemedicine AND Middle East AND Attitude	278
		Telehealth AND MENA AND Barrier	6
		Telemedicine AND MENA AND Barrier	6
		**Total PubMed records that are identified**	**28726**
		**Total records identified from all databases**	**37057**
